# Molecular Diversity of Ectomycorrhizal Fungi in Relation to the Diversity of Neighboring Plant Species

**DOI:** 10.3390/microorganisms12081718

**Published:** 2024-08-20

**Authors:** Weiwei Zhang, Wenyan Xue, Jinliang Liu, Hailan Zhu, Zhong Zhao

**Affiliations:** 1 College of Forestry, Northwest A&F University, Yangling 712100, China; 2 College of Soil and Water Conservation Science and Engineering, Northwest A&F University, Yangling 712100, China

**Keywords:** ectomycorrhizal fungi, plant diversity, neighboring effects, bacterial community, natural secondary forests

## Abstract

(1) Background: Plant diversity has long been assumed to predict soil microbial diversity. The mutualistic symbiosis between forest trees and ectomycorrhizal (EM) fungi favors strong correlations of EM fungal diversity with host density in terrestrial ecosystems. Nevertheless, in contrast with host tree effects, neighboring plant effects are less well studied. (2) Methods: In the study presented herein, we examined the α-diversity, community composition, and co-occurrence patterns of EM fungi in *Quercus acutissima* across different forest types (pure forests, mixed forests with *Pinus tabuliformis*, and mixed forests with other broadleaved species) to ascertain how the EM fungi of focal trees are related to their neighboring plants and to identify the underlying mechanisms that contribute to this relationship. (3) Results: The EM fungal community exhibited an overall modest but positive correlation with neighboring plant richness, with the associations being more pronounced in mixed forests. This neighboring effect was mediated by altered abiotic (i.e., SOC, TN, LC, and LP) and biotic (i.e., bacterial community) factors in rhizosphere soil. Further analysis revealed that *Tomentella_badia*, *Tomentella_galzinii*, and *Sebacina_incrustans* exhibited the most significant correlations with plant and EM fungal diversity. These keystone taxa featured low relative abundance and clear habitat preferences and shared similar physiological traits that promote nutrient uptake through contact, short-distance and medium-distance smooth contact-based exploration types, thereby enhancing the potential correlations between EM fungi and the neighboring plant community. (4) Conclusions: Our findings contribute to the comprehension of the effect of neighboring plants on the EM fungal community of focal trees of different forest communities and the biodiversity sensitivity to environmental change.

## 1. Introduction

The relationship between plants and soil microorganisms provides insights into the ecological drivers of community structure and function. Ectomycorrhizal (EM) fungi constitute a substantial component of the plant–soil microbiome, colonizing up to 95% of the root tips of woody plants [1]. EM symbiotic mutualists benefit the host plant by enhancing nutrient and water uptake from the soil, and the hosts provide fungi with photosynthetic products [2]. Considering the biological functions of the symbiotic process, the correlations between the diversity of EM fungal species and the diversity of EM plant species have been extensively investigated [3,4]. However, the authors of these studies typically collected soil samples that contained mixed roots from co-occurring EM plant species, which potentially overestimates the relationships between EM diversity and host diversity by including the overall positive effect of plant species [5]. In addition, an increasing number of studies have demonstrated that taxonomically and functionally diverse EM fungal communities are associated with the root systems of individual trees [6,7]. Therefore, investigation of the role of individual host species on EM fungal diversity is necessary beyond EM host plant diversity.

Plant species grow in aggregated clumps; a neighborhood of other potential host plants and non-host plants may also affect EM fungi in focal plants. Despite the obvious links, the role of neighboring plant communities in affecting EM fungal communities remains enigmatic. Analogous to the host effects, the diversity of neighboring plants has also been assumed to be positively related to EM fungal diversity, but no direct or negative relationships have also been reported [8,9]. A field investigation into the EM fungal communities of willows (Salix) in an alpine glacier forefield further indicated that the diversity of a plant community only influences the occurrence of a limited number of particular fungi [10]. The neighboring tree effect on focal EM fungal communities warrants improving our understanding of plant mixture impacts on soil processes and competition among microorganisms belowground.

The neighboring effects may be attributed to alterations in abiotic and biotic properties in rhizosphere soil. A number of abiotic factors influence the EM fungal community, with the impact of soil nutrient heterogeneity being particularly noteworthy. These nutrients may be present in the soil solution as soluble inorganic or organic compounds or may be liberated from decomposing organic matter [11]. Leaf litter exhibits significant variation between forest types, and the quality and quantity of leaf litter affects soil microorganisms in both direct and indirect ways. Directly, leaf litter serves as a substrate for soil microorganisms. At the outset of the decomposition process, water-soluble compounds present in the leaf litter are leached into the soil, thereby becoming available for microorganisms [12]. Indirectly, EM fungi are capable of mobilizing nutrients from soil microsites enriched with organic or mineral residues through the secretion of hydrolytic and oxidative enzymes [13]. The alteration in nutrient cycling as a result of litter quality and quantity consequently impacts the population sizes of root-associated microbes by modifying plant–EM interactions [14]. Furthermore, the exchange of nutrients constitutes a critical mechanism through which EM fungi and plants exert reciprocal selective forces on one another. The efficacy of both partners as mutualists may vary. This asymmetry in nutrient exchange has the potential to destabilize EM fungal interactions, as selection favors individuals that provide reduced benefits and incur lower costs [13]. Nevertheless, despite the extensive research on the impact of soil and litter nutrients on the diversity of EM fungi, the underlying mechanisms by which soil nutrient heterogeneity regulates relationships between EM fungal diversity and plant diversity remain unclear.

Alternatively, microbial species in natural ecosystems do not exist in isolation as individual populations; rather, they interact with one another to serve a variety of ecosystem functions [15]. Therefore, our understanding of microbial communities should extend beyond the individual/species-level characteristics of species richness and abundance to encompass the interspecific characteristics of the intricate microbial communities. EM fungi inhabit small pore spaces, where their diminutive size allows for close interactions with rhizosphere soil bacteria, thereby potentially influencing both the composition of the communities and their function [16]. Indeed, evidence suggests that the establishment of EM fungi on tree roots may be influenced by the bacterial communities present in the rhizosphere through the production of cell wall hydrolytic enzymes, phytohormones, amino acids, and/or vitamins [17]. Some of these biochemical activities may exert a direct influence on the germination and growth rate of EM fungal structures, while others may impact root development and susceptibility to infection [17]. Furthermore, it was discovered that interspecies interactions influence the habitat affinities or shared physiologies of microbial community members [18]. These studies have yielded significant insights, indicating that biotic interactions play a pivotal role in shaping microbial communities. Nevertheless, our understanding of the relative importance of environmental heterogeneity and cross-kingdom co-occurrence patterns in the diversity of forest EM fungi and of how bacterial communities regulate the plant–EM fungal diversity relationship remains limited. This represents a key knowledge gap to elucidate the linkages between aboveground and belowground biodiversity, particularly in ecologically fragile areas.

The present study aims to determine how the diversity patterns (abundance, α-diversity index, and compositional dissimilarities) and co-occurrence patterns of EM fungi symbiosed with *Quercus acutissima* are related to the diversity of neighboring plants in three different forest types, i.e., pure forests of *Q. acutissima* (PF), mixed forests of *Q. acutissima* and *Pinus tabuliformis* (QPF), and mixed forests of *Q. acutissima* and other broadleaved species (QBF). The potential mechanisms of neighboring plant diversity effects on EM fungal communities were also determined by incorporating the physiochemical properties (i.e., soil pH, moisture, nutrients, and litter nutrients) and biotic factors (i.e., soil bacterial and cross-kingdom species associations) of rhizosphere soil into the linear mixed-effects model of plant–EM fungal regressions. We sought to address the following questions: (1) how do the EM fungal diversity and co-occurrence patterns of *Q. acutissima* vary across different forest types? Considering the higher resource heterogeneity observed in mixed forests, it is reasonable to hypothesize that EM fungal diversity will be higher in mixed forests than in pure forests. (2) What is the relationship between the EM fungal diversity of focal trees and the diversity of neighboring plants across different forest types? Considering the functional role of soil microbes as the primary decomposers of plant-derived substrates, it is reasonable to hypothesize a positive relationship between them. (3) What environmental factors are responsible for neighboring plant diversity effects on EM fungal communities? The hypothesis is that bacterial community exerts a greater influence on neighboring plant–EM fungal relationships than soil abiotic factors.

## 2. Materials and Methods

### 2.1. Study Area

This research was conducted in the Qiaoshan mountain forest area (35°64′–35°72′ N, 108°91′–109°12′ E, alt 900–1500 m), located in Huangling County of Shaanxi Province in the central and southern of Loess Plateau, China. The study area has a typical warm temperate semiarid climate, with annual mean precipitation of 683.2 mm, an annual mean temperature of 10.2 °C, and a frost-free period of 168 days. Soils are calcareous cinnamon with a clayey texture.

The Qiaoshan mountain has the largest, best-preserved, and most representative natural secondary forest in the central Loess Plateau, which is known as the lungs of the Loess Plateau. According to the historical records from the Qiaoshan Forestry Bureau, the existing natural secondary forests are medium- to old-growth forests dominated by *P. tabulaeformis*, *Q. acutissima*, *Q. wutaishanica*, and *Betula platyphylla* formed naturally from sprouting or seed dispersal since the 1860s when old-growth forests suffered fire, war, and destructive timber harvest.

### 2.2. Experimental Design and Sampling

#### 2.2.1. Sampling Plots and Field Investigation

*Q. acutissima* forests represent the climax community in the study area and are observed in pure forests (PF) or mixed forests with *P. tabuliformis* (QPF) or other broad-leaved oak tree species (QBF). Following a comprehensive field investigation, six independent plots (50 × 50 m) were established at a distance of >3 km for each of the aforementioned communities in August 2020 in mature forests. All selected plots exhibited similar topographies, including elevation (1100–1400 m), slope (18–25°), and aspect (NE-N-NW). Additionally, all plots had not been subject to anthropogenic disturbance since 1965, with the exception of a thinning of 20% intensity in the 1980s.

In each plot, all trees with diameter at breast height (DBH) ≥ 5 cm were identified and their growth status, species name, height, DBH, and crown radius were recorded. Subsequently, five shrubby quadrats, each measuring 5 × 5 m, and five herbaceous quadrats, each measuring 1 × 1 m, were established within each plot for investigation of the species, density, and coverage of shrubs and herbs. Plant richness, defined as the total number of species present in each plot, was selected for further analysis.

#### 2.2.2. Roots and Rhizosphere Soil Sampling

In each of the 18 plots, five healthy and mature trees of *Q. acutissima* were randomly selected, with a minimum distance of 10 m between them, to exclude spatial dependence for most soil and plant variables. These trees were then sampled for first-order roots, which are the most distal and absorptive roots of the branching system, and their rhizosphere soils. The root tracing from the trunk method was employed to trace the first-order root. Specifically, the target individual was identified and excavated to a depth of 20 cm along a main root that could be traced back to the trunk, and the branching fine roots were then picked out. Subsequently, soils adhering to the fine root (diameter < 2 mm) were meticulously brushed after being shaken gently and were thus defined as rhizosphere soils. The root and soil samples collected from the five individuals in each plot were fully homogenized to form one composite sample, resulting in a total of 18 composite samples (*n* = 6 PF; *n* = 6 QBF; *n* = 6 QPF).

The fresh rhizosphere soil samples were sieved through a 2 mm mesh and subsequently divided into three portions for the following analyses: bacterial DNA extraction (stored at −80 °C), microbial biomass and potential extracellular enzymatic activity analysis (stored at 4 °C), and soil physical and chemical property analyses (stored at room temperature). For each root sample, approximately 800 root tips were randomly selected for the detection of EM under the stereomicroscope. Thereafter, the EM root tips were gently washed with sterile distilled water using forceps to remove soil from the surface and then transferred immediately to −80 °C until DNA extraction. 

In addition to the sampling of roots and rhizosphere soil, litter was collected from three 50 × 50 cm quadrats situated beneath the canopy of each selected tree at a distance of approximately 2 m from the trunk. The collected litter samples were subjected to drying at 70 °C and subsequent grinding for nutrient determination.

### 2.3. EM Fungal DNA Extraction and Sequence Processing

EM fungal communities were determined using an Illumina HiSeq 2500 v4 system (Illumina Inc., LC-BIO, Hangzhou, China). Total DNA was extracted from EM root tips using E.Z.N.A.^®^ Soil DNA Kit (Omega Bio-tek, Norcross, GA, USA) and quantified using a NanoDrop ND-2000c UV–Vis spectrophotometer (NanoDrop Technologies Inc., Wilmington, DE, USA) after passing quality control on 2% agarose gels. The fungal communities were characterized by targeting the ITS1 region of the 18S rRNA gene and subsequently amplified through PCR using the primer pair ITS1FI2/ITS2 [19]. The obtained sequences were subjected to quality filtering and subsequently assembled into amplicon sequence variants (ASVs) using DADA2 v1.14 [20]. Taxonomic classification was performed for sequence identification by utilizing the Ribosomal Database Project Classifier tool, which is implemented in the UNITE database. EM fungal species were further identified by referencing the Fungal-Traits Database [21]. All data generated by this study are available at the Sequence Read Archive (SRA) of the National Center for Biotechnology Information (NCBI) under BioProject number PRJNA987964.

### 2.4. EM Fungal Diversity and Network Complexity

The application of different metrics (α diversity, β diversity, network properties, and keystone taxa) allows for the capture of the relations of various dimensions of EM fungal diversity to neighboring plant richness. For α-diversity, the five most frequent indicators, including Shannon, Simpson, ACE, Chao1, and Richness, were calculated. For β-diversity, the total compositional dissimilarities among sites (BetaSOR) of the EM fungal community were partitioned into turnover (BetaSIM) and nestedness (BetaSNE) components based on Sørensen dissimilarity. 

Co-occurrence networks were constructed based on Spearman’s correlation matrices. To ensure the accuracy of taxonomic information, only ASVs with relative abundance ≥ 0.01% were employed in Spearman’s correlation. A series of metrics, including node number and positive and negative edge numbers, were calculated to describe the network topologies. In the constructed networks, the nodes denote individual ASVs, whereas the edges signify robust and statistically significant correlations between these ASVs.

Furthermore, we extracted the topological parameters of individual samples, including node number, edge numbers, and betweenness centrality, using the induced_subgraph function in the R package igraph (https://www.R-project.org/, accessed on 19 November 2022) [22]. Additionally, to summarize the topographical properties of the networks, we computed a single-index multi-complexity (Multi-net) as a synthetic measure by averaging the standardized values of the edge number, node number, and betweenness centrality [23].

The roles of individual nodes were assessed using the within-module connectivity Z, and the among-module connectivity P. Nodes exhibiting high Z- or P-scores were classified as keystone taxa, including peripherals (Zi < 2.5, Pi < 0.62), connectors (Zi < 2.5, Pi > 0.62), network hubs (Zi > 2.5, Pi > 0.62), and module hubs (Zi > 2.5, Pi < 0.62).

### 2.5. Determination of Abiotic and Biotic Factors

Soil pH was quantified in a suspension with a dry soil-to-distilled water ratio of 1:2.5 (*w*/*v*) using a glass electrode. Soil and litter organic carbon (SOC; LC), total nitrogen (TN; LN), and total phosphorus (TP; LP) were determined using the potassium dichromate volumetric method, the Kjeldahl method, and the molybdenum–antimony resistance colorimetric method, respectively. Soil ammonium–nitrogen (NH_4_^+^) and nitrate–nitrogen (NO_3_^−^) were measured using a continuous flow analyzer (AA3, SEAL analytical, Norderstedt, Germany). Soil available P (SAP) was measured using the Olsen method.

DNA extraction, quantification, and sequencing of rhizosphere soil bacteria were conducted using a similar methodology to that employed for root fungal identification, with a few notable distinctions. Specifically, the bacterial community was characterized by targeting the V4–V5 region of the 16S rRNA gene, amplifying it through PCR with the primer pair 515F/907R [24], and taxonomic assignments were made using DADA2 with access to the SILVA database (Release 138).

The topological features of cross-kingdom subnetworks (based on Spearman’s correlation coefficients |r| > 0.6 and *p* < 0.05) were constructed to estimate the potential bacteria effects, which were regarded as biotic factors in examining their contribution to the variation in neighboring plant–EM diversity correlations. The induced biotic factors included bacterial richness (Richness), average degree (AD), average distance (Avgdist), graph density (Den), assortativity (Assort), node number (Vnum), edge numbers (Edgnum), betweenness centrality (Cenbet), degree centrality (Cendeg), and eigenvector centrality (Ceneig).

### 2.6. Statistical Analysis

To evaluate the responses of neighboring plants, abiotic and biotic factors, and EM fungal diversity to forest types, one-way ANOVA followed by a Duncan’s multiple comparisons test (*p* < 0.05) was employed. Changes in each variable induced by forest types were quantified using the natural log-transformed response ratio,$\ln R=\ln\left(X_{E}\right)-\ln\left(X_{C}\right)$, where $X_{E}$ and $X_{C}$ are the mean values of the variables that were observed in the mixed forest and the pure forest, respectively. The variance in lnR was calculated using the following equation: $v=\frac{{\left(SD_{E}\right)}^{2}}{n_{E}{\bar{X}}_{E}^{2}}+\frac{{\left(SD_{C}\right)}^{2}}{n_{C}{\bar{X}}_{C}^{2}}$, where $n_{E}$ and $SD_{E}$ are the sample size and the standard deviation of the mixed forest, respectively, and $n_{C}\;$ and $SD_{C}\;$ are the sample size and the standard deviation of the pure forest, respectively.

Linear regression models were adopted to elucidate the relationship between neighboring plant diversity and EM fungal diversity across various forest types. The Spearman coefficient was utilized to examine the associations between keystone EM fungal taxa and diversity indicators. To mitigate collinearity and streamline the number of explanatory factors, only weakly correlated variables (|r| < 0.6) were retained for further linear mixed-effects models, random forest models, and plspm models, including assortativity, average distance, eigenvector centrality for biotic factors, and SOC, TN, LC, and LP for abiotic factors.

To examine how neighboring plant–EM diversity relationships are influenced by abiotic and biotic variables, a linear mixed-effects model of EM_div_ ∼ Plant_div_ + Env_i_ + Plant_div_ × Env_i_ + (1|Sites) was employed. In these regressions, the response variables were the natural log-transformed EM diversity (Shannon, Simpson, ACE, Richness, Chao1, BetaSOR, BetaSIM, BetaSNE, edge number, node number, betweenness centrality, and multi-complexity), while the independent variable was natural log-transformed neighboring plant diversity. Biotic and abiotic attributes were incorporated as fixed-effect factors affecting both the intercept and the slope (i.e., the interaction between each environmental variable with plant diversity) in models, whereas sites were incorporated as a random effect factor.

To further identify the potential pathways through which biotic and abiotic factors of neighboring plant diversity regulate EM fungal community, we constructed a partial least-squares path model (PLS-PM) [25]. All variables included in the analysis were classified into four groups, including plant diversity (neighboring plant richness), abiotic factors (SOC, TN, LC, and LP), biotic factors (assortativity, average distance, and eigenvector), and EM fungal diversity (α diversity (Shannon, richness, Chao1, ACE, and Simpson), β diversity (the first two axes of PCA), and topological parameters (edge number, node number, betweenness centrality, and multi-complexity). Path coefficients elucidate the directionality and magnitude of the linear associations between latent variables and the explained variance (R^2^). The goodness of fit was used to assess the predictive capacity of the established PLS-PM, with a goodness of fit of >0.5 considered acceptable.

All statistical analyses were conducted in R 4.4.0 using the following packages: vegan for plant richness and EM fungal α diversity; adespatial for β diversity; igraph for network analysis; lme4 for linear mixed-effects models; and plspm for PLS-PM analysis; and ggplot2 for graphical outputs. Co-occurrence networks were visualized using the Gephi platform.

## 3. Results

### 3.1. EM Fungal Diversity under Different Forest Types

**Figure 1:**
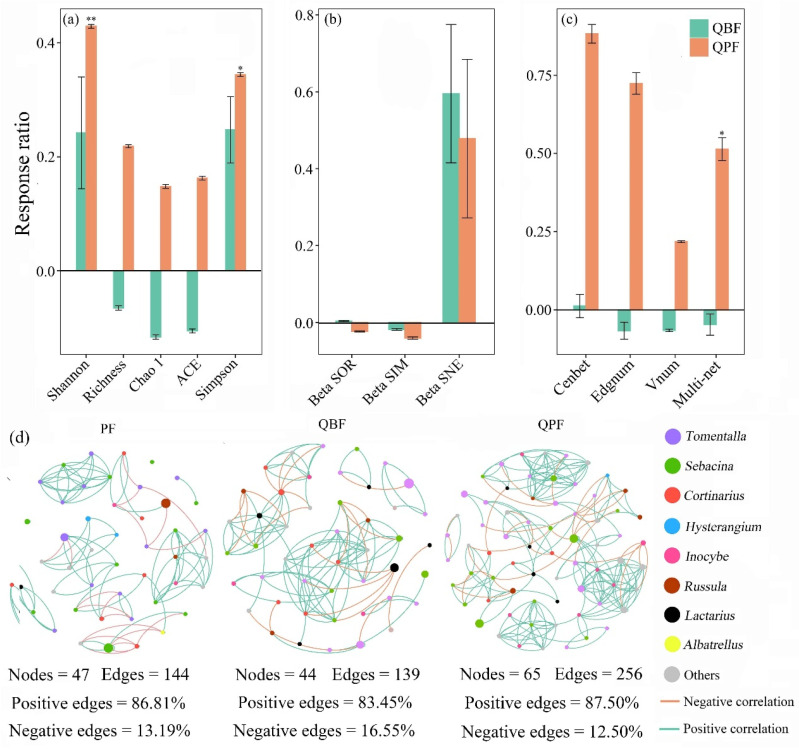
Responses of EM fungal α-diversity (**a**), β- diversity (**b**), and network topological parameters (**c**) to forest types. Response ratios calculated by differences between the variable value in pure forests and those in mixed forests, presented as the means (±SE) of six replicate samples. Statistical significance denoted as * *p* < 0.05, ** *p* < 0.01. (**d**) Visualization of the EM fungal networks for each forest type. Nodes in the constructed network denote individual ASVs colored by genus. Edges represent robust and significant correlations (Spearman’s r > 0.6, *p* < 0.001), divided into positive (green) or negative (orange) edges. QBF, mixed forest of *Q. acutissima* with broad-leaved tree species; QPF, mixed forest of *Q. acutissima* with *P. tabulaeformis*.

The response ratio analysis showed that the EM fungal diversity of *Q. acutissima* was significantly increased in the QPF, with a 41.22% increase in the Simpson index and a 53.58% increase in the Shannon index (Figure 1a). Despite the absence of statistically significant responses to forest types in terms of quantitative β diversity and its turnover and nestedness components (*p* > 0.05), community dissimilarity values were observed to exceed those of pure forests and broad-leaved mixed forests (Figure 1b).

The network of EM fungal communities also exhibited disparate co-occurrence patterns across different forest types. The PF, QBF, and QPF networks captured 144 edges among 47 nodes, 139 edges among 44 nodes, and 256 edges among 65 nodes (Figure 1d). Co-occurrence networks for pure and broad-leaved mixed forests exhibited reduced positive-to-negative edge ratios (PF = 6.58; QBF = 5.04; QPF = 7.00) in comparison with pine–oak forests (Figure 1d). Considering that the positive and negative edges in co-occurrence networks may reflect species cooperation and competition, the lower positive/negative edge ratio probably suggested heightened species competition in pure oak forests. 

Among the subnetworks, the network topological characteristics (betweenness centrality and edge and node number) of EM fungal co-occurrence networks had positive responses to pine mixture, although this was not significant (*p*  > 0.05). The presence of pine neighbors was also found to significantly (*p* < 0.05) enhance the complexity of the EM fungal network of *Q. acutissima* (*p* < 0.05). Nevertheless, the network topological properties and multi-complexity index of the QBF were observed to be lower than those of pure forests (Figure 1c).

### 3.2. Diversity Relationships between EM Fungi and Neighboring Plant Community

**Figure 2:**
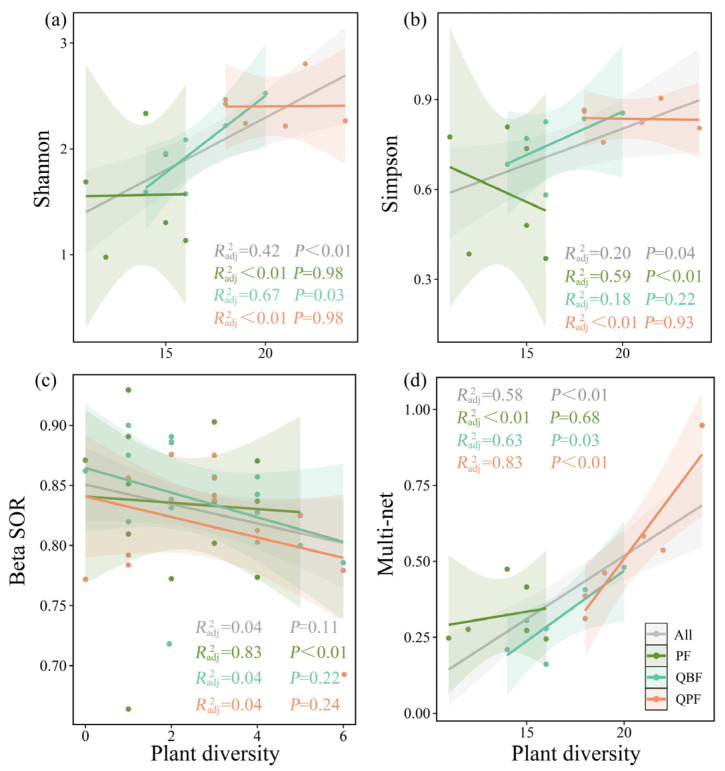
Relationships between neighboring plant diversity and diversity of EM fungal (**a**) Shannon index, (**b**) Simpson index, (**c**) community dissimilarity values and (**d**) community dissimilarity values. The fitted linear models are shown as solid lines, with shading representing 95% confidence intervals. QBF, mixed forest of *Q. acutissima* with broad-leaved tree species; QPF, mixed forest of *Q. acutissima* with *P. tabulaeformis*; PF, pure forest of *Q. acutissima*.

Our study showed overall significant correlations between neighboring plant richness and EM fungal diversity (*R*^2^ = 0.20–0.58, *p* < 0.05; Figure 2 and Figures S2–S4, gray lines), with the exception of EM fungal total dissimilarity (Figure 2c). α diversity, including richness, Shannon, Simpson, ACE, and Chao1 matrices, were modestly but positively correlated to plant diversity (Figure 2a,b and Figure S2, *R*^2^ = 0.20–0.51, *p* < 0.05). Among the metrics of β diversity, the turnover dissimilarity (*R*^2^ = 0.20, *p* < 0.01, Figure S3a) was significantly negatively correlated with plant richness, while nestedness-resultant dissimilarities showed positive correlations (*R*^2^ = 0.40, *p* < 0.01, Figure S3b). The linear regression between network topological features, including betweenness centrality and node and edge numbers, demonstrated a significant positive relationship with plant richness (*R*^2^ = 0.27–0.58, *p* < 0.05, Figure S4). The multi-complexity index, which captures overall network topological properties, was also found to be significantly and positively related to plant diversity (*R*^2^ = 0.58, *p* < 0.01; Figure 2d).

The neighboring plant–EM fungal correlations were found to be influenced by the specific forest types (Figure 2 and Figures S2–S4, green, blue, and red lines). The strongest correlations were generally observed in the QPF. For example, the regression *R*^2^ between plant richness and EM fungal richness was 0.74 in the QPF, which was 1.34 to 1.85 times as strong as in the PF (*R*^2^ = 0.55) and QBF (*R*^2^ = 0.40) (Figure S2a). Similarly, the regression *R^2^* between plant richness and EM fungal network multi-complexity was 0.83 in the QPF, while the values in the PF were <0.01 (Figure 2d). Notably, when examining the Simpson diversity index and Shannon diversity index, QBF showed a relatively higher correlation with plant richness in comparison with the QPF (Figure 2a,b).

### 3.3. Environmental Variables Influencing Diversity Relationships between EM Fungi and the Neighboring Plant Community

We investigated the impacts of biotic and abiotic variables on distinct diversity metrics and topological parameters of EM fungi using slopes of linear mixed-effects regressions (Table 1). The examined environmental variables insignificantly influenced plant–EM fungal relationships in most cases. The total C (SOC and LC) and nutrient content (TN and LP) demonstrated divergent impacts on the slopes and intercepts of diversity relationships. For instance, high EM Shannon and Simpson diversity was associated with lower C content and higher N content for given plant richness. A higher scaling exponent from EM fungal community compositional relationships (total Sørensen dissimilarity and its turnover component) was found to be positively related to LP. Alternatively, slopes of diversity relationship regression were associated positively with assortativity but negatively with the average distance of bacterial networks in most cases (Table 1).

**Figure 3:**
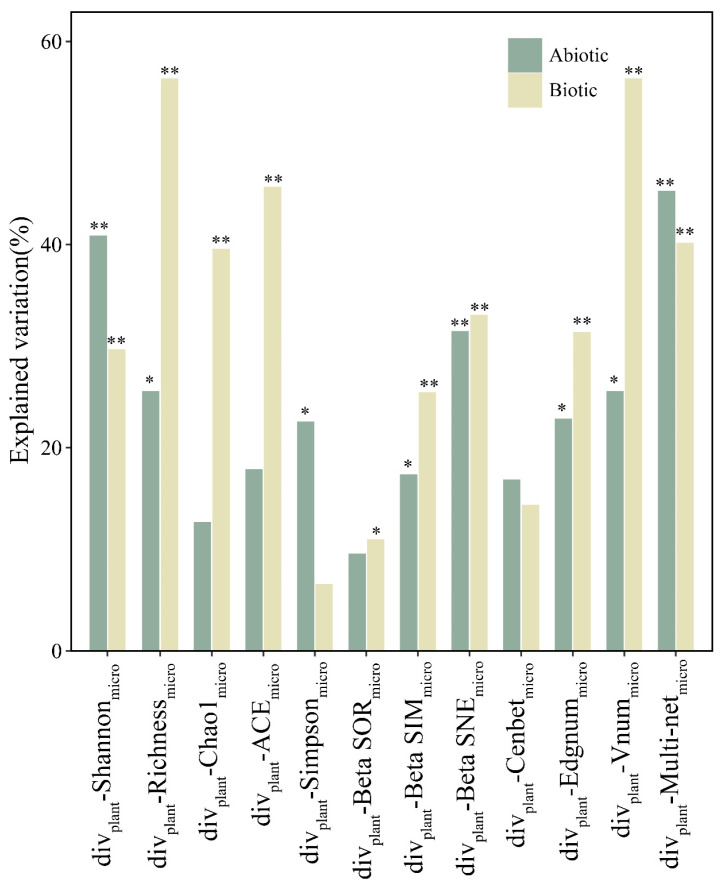
Contributions of biotic and abiotic variables to plant–EM fungal diversity relationships based on the random forest model. Abiotic factors included soil organic C, soil total N, litter organic C, and litter total P. Biotic factors were the topological properties of soil bacterial and cross-kingdom species associations including average distance, eigenvector centrality, and node assortativity coefficients. Significance of the random forest models labelled as * *p* < 0.05, ** *p* < 0.01.

**Figure 4:**
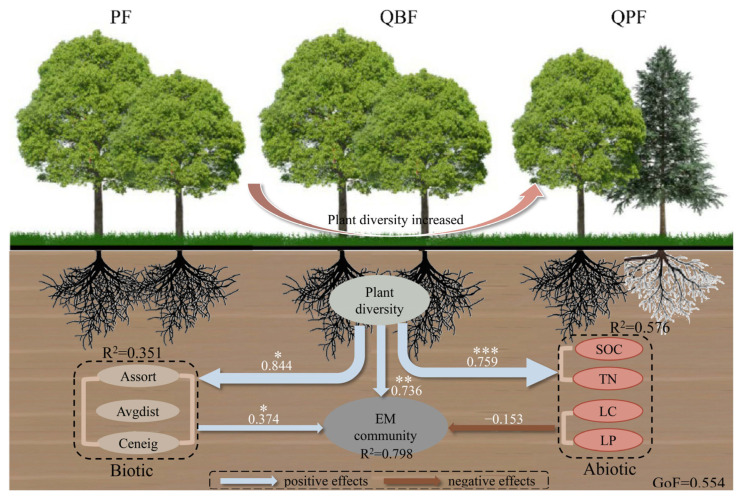
PLS-PM analysis disentangling the mediation effects of abiotic and biotic factors on plant–EM fungal diversity relationships. Solid arrows denote significant relationships, blue arrows signify positive relationships, and brown arrows indicate negative relationships. The numbers are the path coefficients that correspond to the direct effects. *R*^2^ indicates the proportion of variance explained by the variables. GoF represents the proportion of variance accounted for by the model. Plant diversity is community richness including trees, shrubs, and herbs. EM diversity includes α (Richness, Shannon, Simpson, ACE, and Chao1), β (the first two principal co-ordinate components of the Bray–Curtis matrix), and microbial network topology (node number, edge number, betweenness centrality, and multi-complexity index). Significance of the path coefficient denoted with * *p* < 0.05, ** *p* < 0.01, and *** *p* < 0.001.

Random forest models identified the relative contributions of abiotic factors to the relationship between plant and EM communities accounting for 9.5–47.1% of the variability, while the explained variance increased for the effects of biotic factors in most cases (Figure 3). To further explain the pathways through which plant diversity affects EM diversity through biotic and abiotic factors, a robust PLS-PM was constructed (explaining 79.8% of the variance in the EM community) (Figure 4). The results showed that plant diversity was significantly and positively correlated with biotic factors (path coefficients = 0.715) and further significantly and positively correlated with the EM community (path coefficients = −0.574). However, no such correlation was identified between abiotic factors and plant–EM diversity links. The PLS-PM model further confirms that the total effects of biotic factors on the EM community were higher than those of abiotic factors (Figure 4).

### 3.4. Keystone Taxa and Relationships wit the Community Diversity of EM Fungi and Neighboring Plants

A co-occurrence network of higher abundant EM fungi was constructed in order to identify notable species across three forest types. Nine connectors and two module hubs were identified as keystone taxa. Among the identified keystone species, 54.54%, 27.26%, 9.10%, and 9.10% were affiliated with *Tomentella*, *Sebacina*, *Inocybe*, and *Hysterangium* (both members of the phylum Basidiomycota).

**Figure 5:**
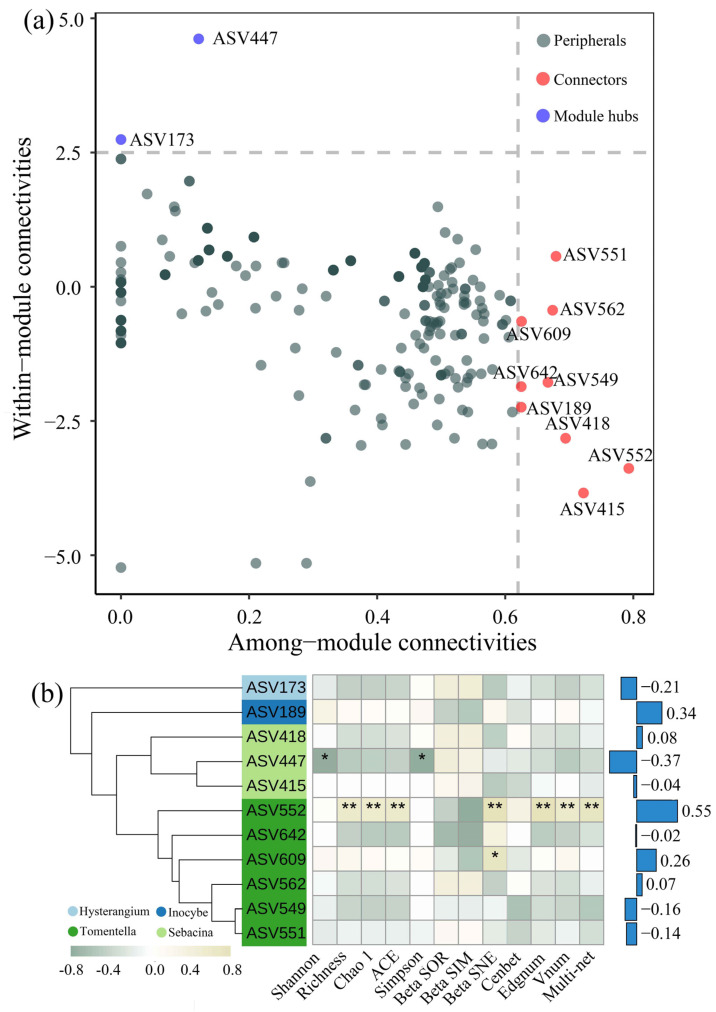
The keystone EM fungal taxa significantly associated with neighboring plant and EM fungal community diversity. (**a**) Zi (within-module connectivity)–Pi (among-module connectivity) plots to identify keystone ASVs within the EM fungal networks. (**b**) Phylogenetic distributions for the keystone taxa. The phylogenetic tree was constructed using the neighbor-joining method. The heatmap of the correlations between keystone ASVs’ abundance and EM fungal diversity, and the bar plot shows the correlations between keystone ASVs and neighboring plant diversity. Values show Spearman’s coefficient and significance levels denoted with * *p* < 0.05, ** *p* < 0.01.

The relative abundance of the keystone ASV552 (*Tomentella_badia*) and ASV609 (*Tomentella_galzinii*) was positively correlated with α diversity (Richness, Chao1, and ACE index), β diversity (BetaSNE), and net topological features of the EM fungal community (*p* < 0.001, Figure 5b). Conversely, the diversity of the EM fungal community increased with the relative abundance of the keystone ASV447 (*Sebacina_incrustans*). Similarly, among all keystone taxa, ASV447 and ASV552 exhibited the most robust yet contrasting correlations with neighboring plant diversity. 

## 4. Discussion

### 4.1. Effects of Forest Types on EM Fungal Diversity

Two antithetic processes, turnover resulting from species replacement and nestedness resulting from species loss, have been demonstrated to drive the responses of microbial community composition to environmental changes [26]. The present study examined turnover and nestedness patterns in the species β diversity of EM fungal communities symbioses with *Q. acutissima* in different forest types and found that the dissimilarity pattern observed in the EM fungal community was predominantly shaped by species turnover (Table S1) caused by spatial constraints or environmental sorting. Similarly, Wu et al. [27] reported significant species turnover patterns for bacteria in lake ecosystems, whereas those of diatoms and chironomids exhibited significant nestedness. The disparate results may be attributed to the distinctive attributes of the investigated taxonomic groups, such as dispersal ability, trophic position, and body size [27,28]. The high spatial turnover of EM fungi also indicates the potential for evolutionary adaptation to environmental conditions across forest types [29]. It is noteworthy that although the contribution of species nestedness in our study was comparable to that found in other regions of the world (usually <20%), the relative importance of the nestedness component was higher in the QPF (17.04%) and QBF (21.89%) than in the PF (13.73%). This may be partially explained by the presence of EM fungal specialists in mixtures of coniferous and other broadleaved tree species, whose loss might contribute to a significant loss of community functions along the environmental heterogeneity. In light of these findings, it can be posited that the loss or gain of species across forest types should also be considered as a significant factor influencing the dynamics of the EM fungal community.

The occurrence patterns of EM fungal communities are also influenced by the forest types. In comparison to pure forests, networks in pine mixture forests exhibited markedly elevated values of betweenness centrality and edge and node numbers in subnetworks and a significantly enhanced multi-complexity index (*p* < 0.05; Figure 1d), which is consistent with hypothesis 1. This finding aligns with prior research indicating that soil fungal networks in mixed forests exhibit elevated topological properties [30]. These alterations in topological parameters, such as more connections and increased centrality, could facilitate the accelerated transmission of external perturbative effects among networked species, thereby enhancing the efficiency of the system [31]. Moreover, the co-occurrence network constitutes a valuable methodology for identifying potential ecological interactions among diverse microbial taxa within the soil across various forest types [32]. The potential EM fungal networks from the PF or QBF exhibited a higher incidence of negative correlations compared to those from the QPF (Figure 1d), indicating increased inter-specific competition within pure forests. Such inter-specific competition could be attributed to the restricted C resources of pure forests, which are typified by sparse vegetation and low primary productivity due to water deficiency. Considering that EM fungal symbiosis is an energy-intensive process, the reduced soil C availability in pure forests may have exerted more significant impacts on potential EM fungal competition than the mixed forest soils. This inference was corroborated by the observation of lower soil water content and nutrient availability in pure forests (Figure S1). Alternatively, the mixed forest provided adequate substrates and diverse habitats for the colonization of EM fungi and facilitated niche differentiation, thereby resulting in a weakening of microbial interactions within the rhizosphere soil [30,33]. 

### 4.2. Effects of Neighboring Plant Community on EM Fungal Diversity

Our study provides evidence for a moderate overall correlation between neighboring plant diversity and the EM fungal community of focal *Q. acutissima* trees with respect to α- diversity, β- diversity, and network topological characteristics (Figure 2 and Figures S2–S4), which is consistent with hypothesis 2. The observed positive relationships provide empirical support for functional interdependence between plants and soil microorganisms at a global scale. An increase in plant diversity can directly affect the diversity patterns of EM fungal communities by altering the amount of photosynthetically derived C allocated to their symbiotic partners [34]. Furthermore, the diverse physiochemical attributes of plant detritus entering the belowground ecosystem could result in the formation of discrete niches for specific decomposers [35]. A plant community with higher diversity may be more productive and provide more resource input, thus serving as more bioavailable substrates (e.g., non-recalcitrant C) and leading to an increase in microbial metabolic activity and potential interactions related to resource utilization and ecological niche differentiation [31]. However, it was noteworthy that compared to the strong correlation between directly interacting host plant and EM fungal diversity, the indirect effects of the neighboring plant community, which encompasses both neighboring host trees, shrubs, and herbs, may be the most plausible explanation for the observed moderate correlation in our study. The presence of other hosts and non-host plants may hamper the development of typical EM fungal communities due to priority effects, allelopathy, and competition among fungi [5,36], leading to a more gradual increase in EM fungal diversity with neighboring plant richness.

When forest types were considered, the EM fungal community showed disparate relationships with neighboring plant diversity (Figure 2). The associations between plant richness and EM fungal diversity were more pronounced in the QPF, whereas in pure forests, plant diversity exhibited a decoupling from Simpson diversity and betweenness centrality. These findings suggest significant roles of tree species composition in the EM fungal symbionts of hosts, thereby influencing the concurrence of both plant and microbial communities. Prior research has indicated that the strength and direction of effects of tree combination on EM fungal communities are associated with plant phylogenetic relationships [37]. Trees with more phylogenetically distant neighbors tend to have higher fungal richness and community dissimilarity compared to trees surrounded by closely related host species [9]. For example, the EM community in *Picea abies* is more sensitive to the presence of *Betula pendula* neighbors than *P. sylvestris* neighbors [4]. Furthermore, the relatively less stressed environments in the QPF may be of particular significance in this context. Forest stands with an admixture of broad-leaved or coniferous tree species contribute to an enhanced variety of root exudates and organic materials, and an abundance of microclimates at floor level and in the soil of forest systems fosters more divergent microhabitats and suitable conditions for a greater variety of EM fungal species in the QPF [38,39]. 

It is increasingly recognized that keystone microbial communities exert significant influences on ecosystem engineering, influencing the assembly and functioning of microbial communities [40]. The Zi-Pi analysis identified eleven keystone EM fungal taxa (nine connectors and two module hubs), mainly belonging to the genera *Tomentella*, *Sebacina*, *Inocybe*, and *Hysterangium* (Figure 5). When examining the relationship between keystone taxa, EM fungal community, and neighboring plant diversity, ASV552 (*Tomentella_badia*), ASV609 (*Tomentella_galzinii*), and ASV447 (*Sebacina_incrustans*) exhibited the most significant correlations. One possible explanation for this result may relate to the physiological traits of these keystone taxa, especially their exploration types, which are commonly assumed to denote the spatial foraging patterns and resource-related niches of extraradical mycelia [41]. Although not all species in a genus were recognized by exploration type in the available records, the genera *Tomentella* and *Sebacina* are generally contact, short-distance, and medium-distance smooth types [42] that have been proposed to maximize the area of hydrophilic hyphae that extend into the soil and promote the rapid uptake of mobile nutrients [43], thereby enhancing the potential correlations with neighboring plant and the EM fungal community. Alternatively, these keystone species are distinguished by their low relative abundance (0.011–0.638%) and distinctive habitat preferences. The results indicate the potential for strong correlations at finer taxonomic resolutions or for specific microbial functional groups, of which the low-abundance microorganisms may be among the key groups that play crucial roles in diversity maintenance [44]. The disproportionate influence of rare taxa is inconsistent with the expected high functional redundancy within microbial communities [44]. The significance of rare taxa may be attributed, at least in part, to the conditional rarity of these microbial communities, which comprise approximately 1.5–28% of all microbes. The conditional rarity indicates that the impact of these microorganisms is subject to external environmental influences and is not a permanent state [45]. Rather, they may serve as repositories of genetic resources that could be mobilized under appropriate conditions [46]. This is reflected in the distinct habitat preferences of these rare taxa. Further research focusing on specific EM fungal functional groups, with clearly identified host plants within the plant community, could significantly enhance the precision of estimates pertaining to plant–microbial relationships.

### 4.3. Environmental Variables Regulate Diversity Relationships between EM Fungi and the Neighboring Plant Community

While it is plausible that soil nutrient heterogeneity facilitates greater diversity of both plant and soil microbial communities, the identified correlations between neighboring plant-EM fungal regression and soil properties in our study do not fully substantiate this perspective. Soil total N was found to be positively correlated with the plant–EM fungal Shannon diversity and Simpson diversity relationship. Similarly, a stronger EM fungal community compositional relationship was observed to be positively correlated with LP (Table 1). However, soil total C was negatively related to the plant–EM fungal Shannon diversity and Simpson diversity relationship, whereas litter total C is the major factor negatively associated with the plant–EM fungal community nestedness dissimilarity correlations. The contrasting correlations of plant–EM fungi relationships to substrate C and nutrient availability may be associated with symbiotic processes between plants and EM fungi [47]. Plants typically allocate 20–40% of their C to EM fungi, which in turn forage for soil nutrients that are not easily accessible to plants [13]. This means that host plants depend on their fungal partners for resource acquisition through a reciprocal exchange of photosynthesis products [2]. The secondary forests under investigation on the Loess Plateau are organic matter-limited ecosystems. The retention and release of other nutrients, such as N and P, by organic matter depends on microbial function and activity [47]. It seems probable that plants growing in these low-organic matter environments formed stronger relationships with microbes through direct symbiosis or nutrient recycling-related feedback [37]. EM fungal colonization can produce nutrient-acquiring enzymes that could probably mine carbon and N from poorly decomposable litters for their own growth and could utilize pine and oak litter as a phosphorous source [48]. This increased availability of nutrients results in an increase in the growth and diversity of plants and soil microorganisms and potential interactions among them.

Although the influence of biotic interaction on biodiversity is a well-documented topic of research, there is comparatively less investigation into the potential implications for the plant–microbial relationship. Our results revealed that plant–EM fungal correlations were significantly related to high assortativity but lower average path distance of the cross-kingdom networks, which indicated a more pronounced influence of coexisting bacterial communities in rhizosphere soil. Further analysis reveals that the biotic factors have more direct and significant effects on EM fungal communities than abiotic factors in natural secondary forests at the southern Loess Plateau (Figure 4), thereby supporting the third hypothesis. Diverse communities are predicted to be more productive than species-poor assemblages due to their enhanced efficiency in capturing limited resources [49]. This has been attributed to niche partitioning allowing for the coexistence of different species by utilizing distinct resources. Furthermore, coexisting species may engage in positive interactions, leading to enhanced community performance [18]. Prior research has indicated that a diverse range of bacteria reside in the surrounding EM symbiosis [50]. These bacteria have been shown to facilitate the establishment of plant–EM fungal symbioses through the stimulation of mycelial extension, increased root–EM fungal colonization, and the mitigation of adverse conditions that impede mycelial spread, referred to as mycorrhiza helper bacteria [17]. These bacterial communities differ from those present in bulk soil and exhibit a greater capacity to solubilize inorganic nutrients compared to non-mycorrhizosphere bacteria [17]. Therefore, the rhizosphere provides a niche for specific bacteria, which, in turn, play an important role in the symbiotic system of trees.

It is now evident that EM fungal diversity significantly impacts a wide array of ecosystem processes, encompassing biogeochemical cycles and eco-evolutionary dynamics within above- and below-ground communities, particularly in the context of global change. Linking plant diversity to EM fungal diversity and soil ecosystem function in field studies is therefore far more challenging, as plant communities are influenced by more than just soil abiotic conditions [17,47]. Our findings provide empirical evidence that rhizosphere bacterial communities significantly and predictably enhance the strength of plant–EM fungi relationships in natural secondary forest ecosystems. It is crucial to acknowledge, however, that association studies do not necessarily attribute the causal effects of plants on the soil community, given the myriad of mechanisms through which the EM fungal community can interact with the plant community [1]. More elaborate indoor experiments under controlled conditions should be conducted to evaluate the relative contributions of species, functional, and genetic diversity in driving these processes, as well as the influence of extrinsic factors in modulating biodiversity–function relationships in future work.

## 5. Conclusions

Our study corroborates significant neighboring plant diversity effects on the EM fungal diversity of focal *Q. acutissima* trees. In general, compared to the strong correlation between directly interacting host plant and EM fungal diversity that has been widely documented, indirect effects of neighboring trees contribute to moderate correlations to EM fungi. EM fungal interactions inferred from network topology (*R*^2^ = 0.27–0.58, *p* < 0.05) showed the strongest correlations with neighboring plant diversity, followed by α-diversity index (*R*^2^ = 0.20–0.51, *p* < 0.05), but the correlation with β diversity was weak and insignificant (*R*^2^ = 0.04, *p* = 0.11). This result indicated insignificant neighboring plant effects on EM fungal community compositional dissimilarity. Mixture forests could enhance the strength of the positive correlation. These results were consistent with the observed increasing EM fungal diversity in mixture forests with *Pinus tabuliformis*, of which the α-diversity index increased by 15.93–53.58%, network complexity-summarized topological parameters increased by 67.72%, while β diversity decreased by 1.2% compared with pure forests. By establishing linkages between environmental heterogeneity and plant-microbe relationships, our analysis revealed the significant roles of high assortativity but lower average path distance of the cross-kingdom networks, indicating that neighboring plants increased EM fungal diversity by increasing the complexity of the coexisting bacterial community in rhizosphere soil. Among the abiotic factors investigated, the slopes of the diversity relationship regression were found to be positively associated with total nutrients (TN and LP) but negatively associated with total C (SOC and LC). These results suggest that the neighboring plant–EM correlation may be more susceptible to environmental fluctuations in systems with high nutrient levels. Overall, our study reveals moderate neighboring plant diversity effects on the EM fungal diversity of *Q. acutissima* in natural secondary forests on the Loess Plateau, pointing to the possibility that this indirect effect of plant diversity may be attributed primarily to altered bacterial communities and nutrients in the rhizosphere soil.

## Figures and Tables

**Table 1:** Linear mixed model of abiotic and biotic variables’ effects on relationships between neighboring plant richness and EM fungal diversity.

		div_plant_- Richness_micro_	div_plant_- Shannon_micro_	div_plant_- Chao1_micro_	div_plant_- ACE _micro_	div_plant_- Simpson_micro_	div_plant_- BetaSOR_micro_	div_plant_- BetaSIM_micro_	div_plant_- BetaSNE_micro_	div_plant_- Cenbet_micro_	div_plant_- Edgnum_micro_	div_plant_- Vnum_micro_	div_plant_- Multi-Net_micro_
General	Intercept	−3447.84	−148.60	2436.40	875.85	−95.02	0.92	0.96	**−0.04**	6.97	−44,888.20	−3447.84	−5.58
	Slope	327.71	9.38	1.82	101.17	6.18	**−0.05**	−0.07	**0.03**	−0.96	3024.03	327.71	−0.27
SOC	Intercept	−17.54	1.89	−32.65	−35.93	0.66	**0.02**	**0.03**	**−0.01**	**0.04**	73.46	−17.54	**0.03**
	Slope	1.02	**−0.10**	1.76	2.00	**−0.03**	**−0.01**	**−0.01**	**0.002**	**−0.003**	−7.51	1.02	−0.003
TN	Intercept	−10.82	−133.13	1612.04	1360.21	−47.59	0.87	0.70	0.17	−7.96	−87,257.70	−10.82	−31.02
	Slope	−15.88	8.18	−103.80	−95.02	2.88	−0.06	−0.15	0.09	0.43	4954.48	−15.88	1.67
LC	Intercept	−0.08	0.15	−1.08	−1.13	**0.05**	**−0.002**	**−0.004**	**0.001**	**−0.01**	32.14	−0.08	**−0.01**
	Slope	**−0.01**	**−0.01**	**0.04**	**0.04**	**−0.002**	**0.001**	**0.002**	**−0.001**	**0.001**	−1.96	**−0.01**	**0.001**
LP	Intercept	−347.77	13.39	−473.94	−567.94	2.95	−0.56	−0.69	0.13	1.14	−10,337.30	−347.77	−0.70
	Slope	21.62	−0.90	29.20	35.16	−0.21	0.18	0.25	−0.07	−0.07	614.12	21.61	**0.03**
Avgdist	Intercept	−1.40	−0.49	11.70	8.30	−0.29	**0.03**	**0.01**	**0.01**	**0.004**	−897.31	−1.40	−0.15
	Slope	0.67	**0.03**	−0.11	0.10	**0.02**	**−0.003**	**−0.001**	**−0.001**	**−0.001**	54.88	0.67	**0.01**
Ceneig	Intercept	4145.07	132.09	−2046.12	−158.27	92.64	−8.52	−6.67	−1.85	−1.29	89,378.99	4145.08	26.95
	Slope	−358.01	−8.88	−10.86	−128.64	−6.22	2.12	1.70	0.43	0.63	−5480.61	−358.01	−0.92
Assott	Intercept	88.19	−2.70	157.25	155.07	−0.74	**0.001**	**−0.02**	**0.02**	−0.41	1110.58	88.19	−0.32
	Slope	−6.13	0.17	−10.03	−10.03	**0.04**	**0.01**	**0.02**	**−0.01**	**0.03**	−61.72	−6.13	**0.03**
Random effects (variance)													
Sites		1.82	0.10	5.21	4.95	**0.03**	**0.04**	**0.05**	**0.02**	**0.03**	196.62	1.82	0.07
residual		0.68	**0.04**	1.95	1.85	**0.01**	**0.02**	**0.02**	**0.01**	**0.01**	73.73	0.68	**0.03**

For each EM fungal diversity index, a linear mixed-effects model of EM_div_ ∼ Plant_div_ + Env_i_ + Plant_div_ × Env_i_ + (1|Sites) was performed, where fixed effects refers to biotic (topological properties of soil bacterial and cross-kingdom species associations including average distance, eigenvector centrality, and node assortativity coefficients) and abiotic (soil organic C, soil total N, litter organic C, and litter total P) variables. Significant effects of fixed-effect factors are given in bold.

## Data Availability

The data presented in this study are available on request from the corresponding author due to the project has not yet been finished.

## Supplementary Materials

The following supporting information can be downloaded at: https://www.mdpi.com/article/10.3390/microorganisms12081718/s1. Figure S1: Responses of abiotic and biotic variables to forest types. Response ratios calculated by differences between the variable value in pure forests and those in mixed forests, presented as the means (±SE) of six replicate samples. Statistical significance denoted as * *p* < 0.05, ** *p* < 0.01, and *** *p* < 0.001. pH, soil pH value; SWC, soil water content; SOC, soil organic C; TN, soil total N; TP, soil total P; NH_4_^+^, soil ammonium-N; NO_3_^−^, soil nitrate-N; SAP, soil available P; LC, litter organic C; LN, litter total N; LP, litter total P; Richness, bacterial richness; AD, average degree; Avgdist, average distance; Cenbet, betweenness centrality; Cendeg, degree centrality; Ceneig, eigenvector centrality; Den, graph density; Assort, assortativity; Edgnum, edge number; Vnum, node number; APD, average path distance; QBF, mixed forest of *Q. acutissima* with broad-leaved tree species; QPF, mixed forest of *Q. acutissima* with *Pinus tabulaeformis*; PF, pure forest of *Q. acutissima*. Figure S2: Relationships between neighboring plant diversity and EM fungal α-diversity. The fitted linear models are shown as solid lines, with shading representing 95% confidence intervals. QBF, mixed forest of *Q. acutissima* with broad-leaved tree species; QPF, mixed forest of *Q. acutissima* with *P. tabulaeformis*; PF, pure forest of *Q. acutissima*. Figure S3: Relationships between neighboring plant diversity and EM fungal β-diversity. The fitted linear models are shown as solid lines, with shading representing 95% confidence intervals. QBF, mixed forest of *Q. acutissima* with broad-leaved tree species; QPF, mixed forest of *Q. acutissima* with *P. tabulaeformis*; PF, pure forest of *Q. acutissima.* Figure S4: Relationships between neighboring plant diversity and EM fungal network topology. The fitted linear models are shown as solid lines, with shading representing 95% confidence intervals. QBF, mixed forest of *Q. acutissima* with broad-leaved tree species; QPF, mixed forest of *Q. acutissima* with *P. tabulaeformis*; PF, pure forest of *Q. acutissima*. Table S1: Comparisons of β diversity in all samples of different forest types.

## References

[1] Linde S., Suz L.M., Orme C.D.L., Cox F., Andreae H., Asi Endla Atkinson B., Benham S., Carroll C., Cools N., De Vos B. (2018). Environment and host as large-scale controls of ectomycorrhizal fungi. Nature.

[2] Bogar L.M., Tavasieff O.S., Raab T.K., Peay K.G. (2022). Does resource exchange in ectomycorrhizal symbiosis vary with competitive context and nitrogen addition?. New Phytol..

[3] Reis F., Valdiviesso T., Varela C., Tavares R.M., Baptista P., Lino-Neto T. (2018). Ectomycorrhizal fungal diversity and community structure associated with cork oak in different landscapes. Mycorrhiza.

[4] Otsing E., Anslan S., Ambrosio E., Koricheva J., Tedersoo L. (2021). Tree species richness and neighborhood effects on ectomycorrhizal fungal richness and community structure in boreal forest. Front. Microbiol..

[5] Tedersoo L., Bahram M., Dickie I.A. (2014). Does host plant richness explain diversity of ectomycorrhizal fungi? Re-evaluation of Gao et al. (2013) data sets reveals sampling effects. Mol. Ecol..

[6] Mandolini E., Bacher M., Peintner U. (2022). Ectomycorrhizal fungal communities of Swiss stone pine (*Pinus cembra*) depend on climate and tree age in natural forests of the Alps. Plant Soil.

[7] Yang T., Song L.Y., Lin H.Y., Dong K., Fu X., Gao G.F., Adams J.M., Chu H.Y. (2023). Within-species plant phylogeny drives ectomycorrhizal fungal community composition in tree roots along a timberline. Soil Biol. Biochem..

[8] Liu L., Zhu K., Wurzburger N., Zhang J. (2020). Relationships between plant diversity and soil microbial diversity vary across taxonomic groups and spatial scales. Ecosphere.

[9] Nguyen N.H., Williams L.J., Vincent J.B., Stefanski A., Cavender-Bares J., Messier C., Paquette A., Gravel D., Reich P.B., Kennedy P.G. (2016). Ectomycorrhizal fungal diversity and saprotrophic fungal diversity are linked to different tree community attributes in a field-based tree experiment. Mol. Ecol..

[10] Arraiano-Castilho R., Bidartondoet M.I., Niskanen T., Zimmermann S., Frey B., Brunner I., Senn-Irlet B., Hörandl E., Gramlich S., Suz L.M. (2020). Plant-fungal interactions in hybrid zones: Ectomycorrhizal communities of willows (Salix) in an alpine glacier forefield. Fungal Ecol..

[11] Qualls R.G. (2000). Comparison of the behavior of soluble organic and inorganic nutrients in forest soils. For. Ecol. Manag..

[12] Xu J.W., Lin G., Liu B., Mao R. (2020). Linking leaf nutrient resorption and litter decomposition to plant mycorrhizal associations in boreal peatlands. Plant Soil.

[13] Tunlid A., Floudas D., Koide R., Rineau F. (2016). Soil organic matter decomposition mechanisms in ectomycorrhizal fungi. Molecular Mycorrhizal Symbiosis.

[14] Aponte C., García L.V., Marañón T., Gardes M. (2010). Indirect host effect on ectomycorrhizal fungi: Leaf fall and litter quality explain changes in fungal communities on the roots of co-occurring Mediterranean oaks. Soil Biol. Biochem..

[15] Tu Q., Yan Q., Deng Y., Michaletz S.T., Buzzard V., Weiser M.D., Waide R., Ning D., Wu L., He Z. (2020). Biogeographic patterns of microbial co-occurrence ecological networks in six American forests. Soil Biol. Biochem..

[16] Burke D.J., Carrino-Kyker S.R. (2021). The influence of mycorrhizal fungi on rhizosphere bacterial communities in forests. For. Microbiol..

[17] Deveau A., Labbé J. (2016). Mycorrhiza helper bacteria. Mol. Mycorrhizal Symbiosis.

[18] Kennedy P. (2010). Ectomycorrhizal fungi and interspecific competition: Species interactions, community structure, coexistence mechanisms, and future research directions. New Phytol..

[19] Karlsson I., Friberg H., Steinberg C., Persson P. (2014). Fungicide effects on fungal community composition in the wheat phyllosphere. PLoS ONE.

[20] Callahan B.J., McMurdie P.J., Rosen M.J., Han A.W., Johnson A.J.A., Holmes S.P. (2016). DADA2: High-resolution sample inference from Illumina amplicon data. Nat. Methods.

[21] Põlme S., Abarenkov K., Nilsson R.H., Lindahl B.D., Clemmensen K.E., Kauserud H., Nguyen N., Kjøller R., Bates S.T., Baldrian P. (2020). FungalTraits: A user-friendly traits database of fungi and fungus-like stramenopiles. Fungal Divers..

[22] Csardi G., Tamas N. (2006). The igraph software package for complex network research. Interj. Complex Syst..

[23] Hu A., Wang J., Sun H., Niu B., Si G., Wang J., Yeh C.F., Zhu X., Lu X., Zhou J. (2020). Mountain biodiversity and ecosystem functions: Interplay between geology and contemporary environments. ISME J..

[24] Degnan P.H., Ochman H. (2012). Illumina-based analysis of microbial community diversity. ISME J..

[25] Sanchez G., Trinchera L., Russolillo G. (2013). Package ‘Plspm’.

[26] Wang X., Zhong M., Yang S., Jiang J., Hu J. (2022). Multiple β-diversity patterns and the underlying mechanisms across amphibian communities along a subtropical elevational gradient. Divers. Distrib..

[27] Wu K., Zhao W., Li M., Picazo F., Soininen J., Shen J., Zhu L., Cheng X., Wang J. (2020). Taxonomic dependency of beta diversity components in benthic communities of bacteria, diatoms and chironomids along a water-depth gradient. Sci. Total Environ..

[28] Wang J., Soininen J., Zhang Y., Wang B., Yang X., Shen J. (2012). Patterns of elevational beta diversity in micro-and macroorganisms. Glob. Ecol. Biogeogr..

[29] Wagstaff M.C., Howell K.L., Bett B.J., Billett D.S.M., Brault S., Stuart C.T., Rex M.A. (2014). β-diversity of deep-sea holothurians and asteroids along a bathymetric gradient (NE Atlantic). Mar. Ecol. Prog. Ser..

[30] Huo X., Ren C., Wang D., Wu R., Wang Y., Li Z., Huang D., Qi H. (2023). Microbial community assembly and its influencing factors of secondary forests in Qinling Mountains. Soil Biol. Biochem..

[31] Wu H., Gao T., Hu A., Wang J. (2024). Network Complexity and Stability of Microbes Enhanced by Microplastic Diversity. Environ. Sci. Technol..

[32] Lei S., Wang X., Wang J., Zhang L., Liao L., Liu G., Wang G., Song Z., Zhang C. (2024). Effect of aridity on the β-diversity of alpine soil potential diazotrophs: Insights into community assembly and co-occurrence patterns. mSystems.

[33] Zhang X., Huang Y., Liu S., Fu S., Ming A., Li X., Yao M., Li H., Tian C. (2019). Mixture of tree species enhances stability of the soil bacterial community through phylogenetic diversity. Eur. J. Soil Sci..

[34] Johnson D., IJdo M., Genney D.R., Anderson I.C., Alexanderet I.J. (2005). How do plants regulate the function, community structure, and diversity of mycorrhizal fungi?. J. Exp. Bot..

[35] Heděnec P., Zheng H., Siqueira D.P., Lin Q., Peng Y., Schmidt I.K., Frøslev T.D., Kjøller R., Rousk J., Vesterdal L. (2023). Tree species traits and mycorrhizal association shape soil microbial communities via litter quality and species mediated soil properties. For. Ecol. Manag..

[36] Garrido J.L., Alcántara J.M. (2022). Scale dependency of ectomycorrhizal fungal community assembly processes in Mediterranean mixed forests. Mycorrhiza.

[37] Tedersoo L., Bahram M. (2019). Mycorrhizal types differ in ecophysiology and alter plant nutrition and soil processes. Biol. Rev..

[38] Gillespie L.M., Hättenschwiler S., Milcu A., Wambsganss J., Shihan A., Fromin N. (2021). Tree species mixing affects soil microbial functioning indirectly via root and litter traits and soil parameters in European forests. Funct. Ecol..

[39] Smolander A., Kitunen V. (2021). Soil organic matter properties and C and N cycling processes: Interactions in mixed-species stands of silver birch and conifers. Appl. Soil Ecol..

[40] Liu S., Yu H., Yu Y., Huang J., Zhou Z., Zeng J., Chen P., Xiao F., He Z., Yan Q. (2022). Ecological stability of microbial communities in Lake Donghu regulated by keystone taxa. Ecol. Indic..

[41] Hobbie E.A., Agerer R. (2010). Nitrogen isotopes in ectomycorrhizal sporocarps correspond to belowground exploration types. Plant Soil.

[42] Agerer R. (2001). Exploration types of ectomycorrhizae: A proposal to classify ectomycorrhizal mycelial systems according to their patterns of differentiation and putative ecological importance. Mycorrhiza.

[43] Jörgensen K., Clemmensen K.E., Wallander H., Lindahl B.D. (2023). Do ectomycorrhizal exploration types reflect mycelial foraging strategies?. New Phytol..

[44] Dawson W., Hör J., Egert M., Kleunen M., Pester M. (2017). A small number of low-abundance bacteria dominate plant species-specific responses during rhizosphere colonization. Front. Microbiol..

[45] Liang Y., Xiao X., Nuccio E.E., Yuan M., Zhang N., Xue K., Cohan F.M., Zhou J., Sun B. (2020). Differentiation strategies of soil rare and abundant microbial taxa in response to changing climatic regimes. Environ. Microbiol..

[46] Wan W., Grossart H.P., He D., Yuan W., Yang Y. (2021). Stronger environmental adaptation of rare rather than abundant bacterioplankton in response to dredging in eutrophic Lake Nanhu (Wuhan, China). Water Res..

[47] Liu Y., Li X., Kou Y. (2020). Ectomycorrhizal fungi: Participation in nutrient turnover and community assembly pattern in forest ecosystems. Forests.

[48] Kuyper T.W., Suz L.M. (2023). Do ectomycorrhizal trees select ectomycorrhizal fungi that enhance phosphorus uptake under nitrogen enrichment?. Forests.

[49] Becker J., Eisenhauer N., Scheu S., Jousset A. (2012). Increasing antagonistic interactions cause bacterial communities to collapse at high diversity. Ecol. Lett..

[50] Haq I.U., Zhang M., Yang P., Elsas J.D. (2014). The interactions of bacteria with fungi in soil: Emerging concepts. Adv. Appl. Microbiol..

