# Supplementary material for: Molecular Diversity of Ectomycorrhizal Fungi in Relation to the Diversity of Neighboring Plant Species
Source: Microorganisms. 2024 Aug 20;12(8):1718. doi: 10.3390/microorganisms12081718 (PMC11356974; doi:10.3390/microorganisms12081718)
Supplement: Supplementary file 1 [file microorganisms-12-01718-s001.zip › microorganisms-3151625-supplementary.pdf]

Figure S1. Responses of abiotic and biotic variables to forest types. Response ratios calculated by differences between the variable value in pure forests and that in mixed forests, presented as the means (±SE) of six replicate samples. Statistical significance denoted as *$P < 0.05$, **$P < 0.01$, and *** $P < 0.001$. pH, soil pH value; SWC, soil water content; SOC, soil organic C; TN, soil total N; TP, soil total P; NH_4^+, soil ammonium-N; NO_3^-, soil nitrate-N; SAP, soil available P; LC, litter organic C; LN, litter total N; LP, litter total P; Richness, bacterial richness; AD, average degree; Avgdist, average distance; Cenbet, betweenness centrality; Cendeg, degree centrality; Ceneig, eigenvector centrality; Den, graph density; Assort, assortativity; Edgnum, edge numbers; Vnum, node numbers; APD, average path distance. QBF, mixed forest of *Q. acutissima* with broad-leaved tree species; QPF, mixed forest of *Q. acutissima* with P*inus tabulaeformis*; PF, pure forest of *Q. acutissima*.

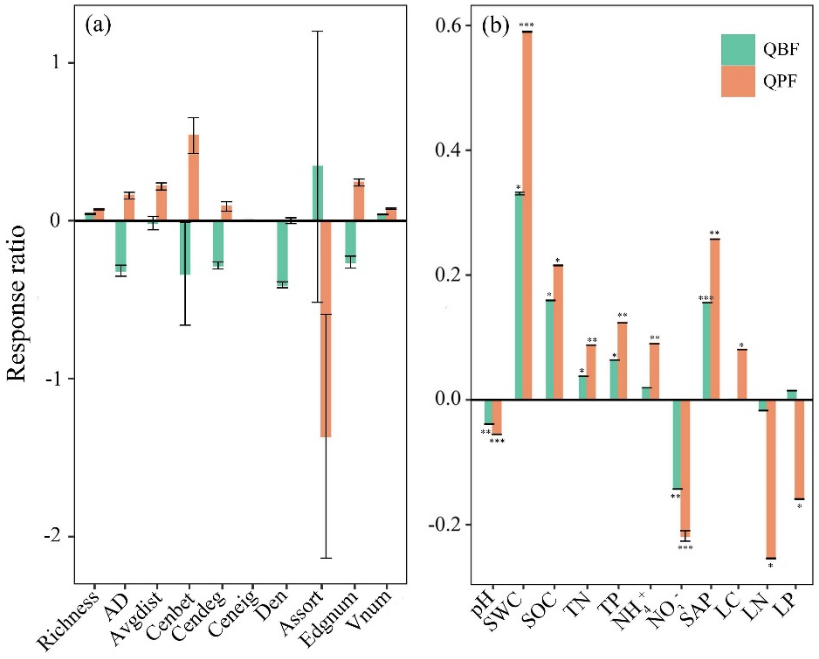

Figure S2. Relationships between neighboring plant diversity and EM fungal α-diversity. The

fitted linear models are shown as solid lines, with shading representing 95% confidence intervals. QBF, mixed forest of *Q. acutissima* with broad-leaved tree species; QPF, mixed forest of *Q. acutissima* with *P. tabulaeformis*; PF, pure forest of *Q. acutissima*. **P* < 0.05, ***P* < 0.01 and *** *P* < 0.001.

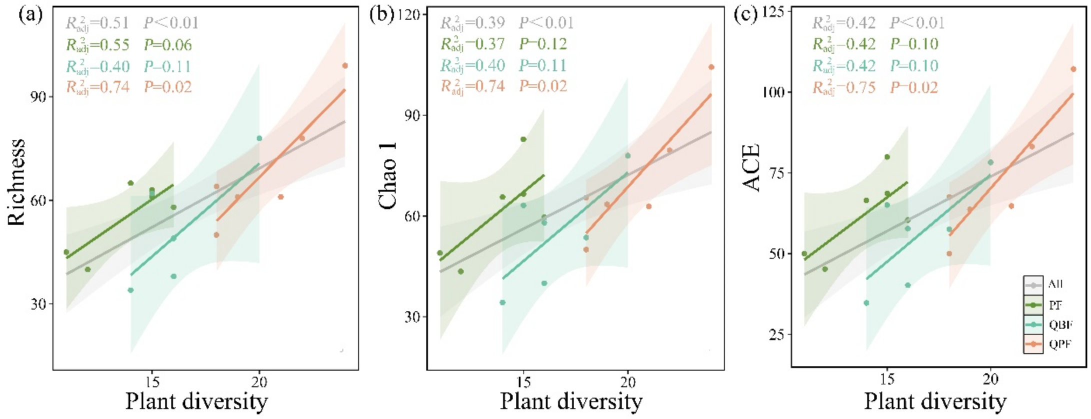

Figure S3. Relationships between neighboring plant diversity and EM fungal β-diversity. The fitted linear models are shown as solid lines, with shading representing 95% confidence intervals. QBF, mixed forest of *Q. acutissima* with broad-leaved tree species; QPF, mixed forest of *Q. acutissima* with *P. tabulaeformis*; PF, pure forest of *Q. acutissima*. *$P < 0.05$, **$P < 0.01$, and *** $P < 0.001$.

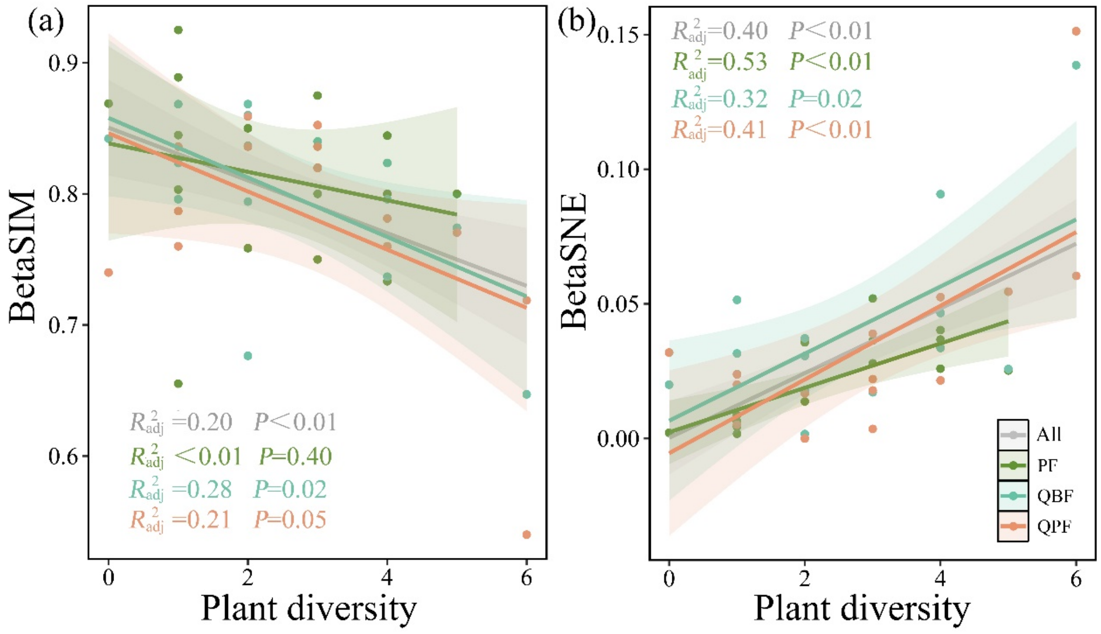

Figure S4. Relationships between neighboring plant diversity and EM fungal network topology. The fitted linear models are shown as solid lines, with shading representing 95% confidence intervals. QBF, mixed forest of *Q. acutissima* with broad-leaved tree species; QPF, mixed forest of *Q. acutissima* with *P. tabulaeformis*; PF, pure forest of *Q. acutissima*. **P* < 0.05, ***P* < 0.01, and *** *P* < 0.001.

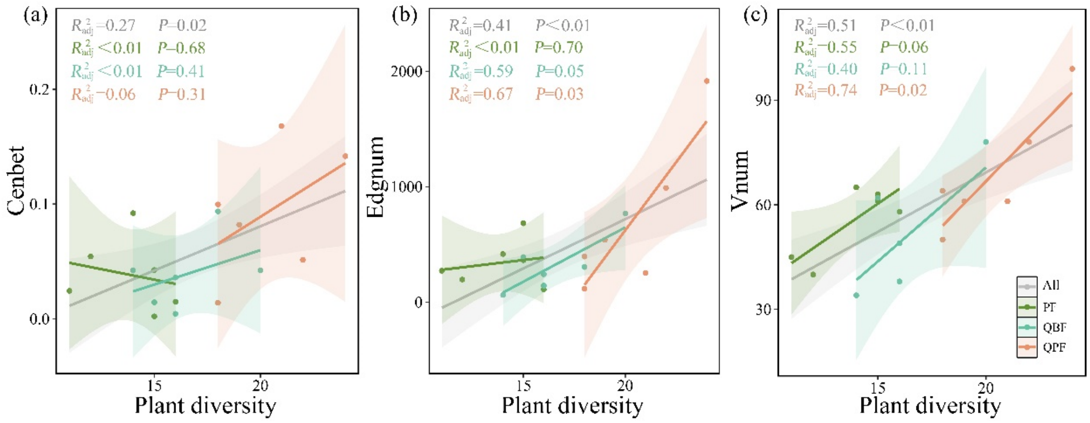

Table S1. Comparisons of β-diversity in all samples of different forest types.

	Beta SIM/Beta SOR	Beta SNE/Beta SOR
PF	0.974	0.026
QBF	0.953	0.047
QPF	0.957	0.043
Whole	0.960	0.040
